# Interdisziplinäres, kollaboratives D-A-CH Konsensus-Statement zur Diagnostik und Behandlung von Myalgischer Enzephalomyelitis/Chronischem Fatigue*-*Syndrom

**DOI:** 10.1007/s00508-024-02372-y

**Published:** 2024-05-14

**Authors:** Kathryn Hoffmann, Astrid Hainzl, Michael Stingl, Katharina Kurz, Beate Biesenbach, Christoph Bammer, Uta Behrends, Wolfgang Broxtermann, Florian Buchmayer, Anna Maria Cavini, Gregory Sacha Fretz, Markus Gole, Bettina Grande, Tilman Grande, Lotte Habermann-Horstmeier, Verena Hackl, Jürg Hamacher, Joachim Hermisson, Martina King, Sonja Kohl, Sandra Leiss, Daniela Litzlbauer, Herbert Renz-Polster, Wolfgang Ries, Jonas Sagelsdorff, Carmen Scheibenbogen, Bernhard Schieffer, Lena Schön, Claudia Schreiner, Kevin Thonhofer, Maja Strasser, Thomas Weber, Eva Untersmayr

**Affiliations:** 1 Allgemeinmedizin, Public Health und Versorgungsforschung, Abteilung für Primary Care Medicine, Zentrum für Public Health, Medizinische Universität Wien, Kinderspitalgasse 15, 1090 Wien, Österreich; 2 Österreichische Gesellschaft für ME/CFS, Wien, Österreich; 3 Neurologie, Facharztzentrum Votivpark, Wien, Österreich; 4 Innere Medizin, Universitätsklinik für Innere Medizin II, MedUni Innsbruck, Innsbruck, Österreich; 5 Kinder- und Jugendheilkunde, kokon – Reha für junge Menschen, Kinder-Reha Rohrbach-Berg GmbH, Rohrbach-Berg, Österreich; 6 Innere Medizin, Nephrologie & Geriatrie, a. ö. BKH Kufstein, Kufstein, Österreich; 7 MRI Chronische Fatigue Centrum für junge Menschen (MCFC), Zentrum für Kinder- und Jugendmedizin: eine Kooperation des Klinikums rechts der Isar, Technischen Universität München und der München Klinik gGmbH, München, Deutschland; 8 Neuropädiatrie und Sozialpädiatrie, Kempfeld, Deutschland; 9 Psychiatrie und Psychotherapie, Abteilung für Psychiatrie und Psychotherapie, Krankenhaus der Barmherzigen Brüder, Eisenstadt, Österreich; 10 Fachärztin für Kinder- und Jugendheilkunde, Psychotherapeutische Medizin, St.Veit/Glan, Österreich; 11 Department Innere Medizin, Medizinische Poliklinik, Kantonsspital Graubünden, Loestraße 170, 7000 Chur, Schweiz; 12 Psychologie und Philosophie, Praxis für Psychologie, Philosophie und Berufskunde, Linz, Österreich; 13 Psychotherapie und Psychoanalyse, Heidelberg, Deutschland; 14 Villingen Institute of Public Health (VIPH), Villingen-Schwenningen, Deutschland; 15 Physiotherapie, AUVA Rehabilitationszentrum Meidling, Wien, Österreich; 16 Innere Medizin und Pneumologie, Lindenhofspital, Bern, Schweiz; 17 Biomathematik, Fakultät für Mathematik, Universität Wien, Wien, Österreich; 18 Department of Structural and Computational Biology, Max Perutz Labs, Wien, Österreich; 19 Lehrstuhl für Medical Humanities, Mathematisch-Naturwissenschaftliche und Medizinische Fakultät, Universität Fribourg, Fribourg, Schweiz; 20 #MillionsMissing Deutschland, Bedburg-Hau, Deutschland; 21 HNO, St. Valentin, Niederösterreich Österreich; 22 Kinder- und Jugendheilkunde, Zentrum für Präventivmedizin und Digitale Gesundheit, Abteilung Allgemeinmedizin, Universitätsmedizin Mannheim, Universität Heidelberg, Heidelberg, Deutschland; 23 Nephrologie, Dialyse, DIAKO Krankenhaus gGmbH, Flensburg, Deutschland; 24 Schweizerische Gesellschaft für ME & CFS, Zürich, Schweiz; 25 Institut für Med. Immunologie, Sektion Immundefekte und Postinfektiöse Erkrankungen, Charité – Universitätsmedizin Berlin, Campus Virchow-Klinikum, Berlin, Deutschland; 26 Klinik für Innere Medizin-Kardiologie- Angiologie und Internistische Intensivmedizin und Zentrums für Notfallmedizin, Universitätsklinikum Gießen und Marburg GmbH, Standort Marburg, Marburg, Deutschland; 27 Physiotherapie, Physio Austria: Fachgruppe für komplexe Multisystemerkrankungen, Wien, Österreich; 28 Neurologie, Neurologische Praxis Solothurn, Solothurn, Schweiz; 29 Schmerzmedizin, Facharzt für Anästhesie und Intensivmedizin, Graz, Österreich; 30 Klinische Immunologie, Institut für Pathophysiologie und Allergieforschung, Zentrum für Pathophysiologie, Infektiologie und Immunologie, Medizinische Universität Wien, Wien, Österreich

**Keywords:** ME/CFS, D‑A-CH-Konsensusstatement, Kanadische Konsensuskriterien, Leitsymptom post-exertionelle Malaise, ME/CFS, Consensus statement, Post-Exertional Malaise, Diagnostics, Therapy

## Abstract

Myalgische Enzephalomyelitis/Chronisches Fatigue-Syndrom (ME/CFS) ist eine schwere, chronische Multisystemerkrankung, die je nach Ausprägung zu erheblichen körperlichen und kognitiven Einschränkungen, zum Verlust der Arbeitsfähigkeit bis hin zur Pflegebedürftigkeit einschließlich künstlicher Ernährung und in sehr schweren Fällen sogar zum Tod führen kann. Das Ziel dieses D-A-CH-Konsensusstatements ist es, 1) den aktuellen Wissensstand zu ME/CFS zusammenzufassen, 2) in der Diagnostik die kanadischen Konsensuskriterien (CCC) als klinische Kriterien mit Fokus auf das Leitsymptom post-exertionelle Malaise (PEM) hervorzuheben und 3) vor allem im Hinblick auf Diagnostik und Therapie einen Überblick über aktuelle Optionen und mögliche zukünftige Entwicklungen aufzuzeigen. Das D-A-CH-Konsensusstatement soll Ärzt:innen, Therapeut:innen und Gutachter:innen dabei unterstützen, Patient:innen mit Verdacht auf ME/CFS mittels adäquater Anamnese und klinisch-physikalischen Untersuchungen sowie der empfohlenen klinischen CCC zu diagnostizieren und dabei die präsentierten Fragebögen sowie die weiteren Untersuchungsmethoden zu nutzen. Der Überblick über die zwei Säulen der Therapie bei ME/CFS, Pacing und die symptomlindernden Therapieoptionen sollen nicht nur Ärzt:innen und Therapeut:innen zur Orientierung dienen, sondern auch Entscheidungsträger:innen aus der Gesundheitspolitik und den Versicherungen darin unterstützen, welche Therapieoptionen bereits zu diesem Zeitpunkt bei der Indikation „ME/CFS“ von diesen erstattbar sein sollten.

## Abstract (en)

Myalgic encephalomyelitis/chronic fatigue syndrome (ME/CFS) is a severe, chronic multisystemic disease which, depending on its severity, can lead to considerable physical and cognitive impairment, loss of ability to work and the need for nursing care including artificial nutrition and, in very severe cases, even death.The aim of this D-A-CH (Germany, Austria, Switzerland) consensus statement is 1) to summarize the current state of knowledge on ME/CFS, 2) to highlight the Canadian Consensus Criteria (CCC) as clinical criteria for diagnostics with a focus on the leading symptom post-exertional malaise (PEM) and 3) to provide an overview of current options and possible future developments, particularly with regard to diagnostics and therapy. The D-A-CH consensus statement is intended to support physicians, therapists and valuer in diagnosing patients with suspected ME/CFS by means of adequate anamnesis and clinical-physical examinations as well as the recommended clinical CCC, using the questionnaires and other examination methods presented. The overview of the two pillars of therapy for ME/CFS, pacing and symptom-relieving therapy options, is intended not only to provide orientation for physicians and therapists, but also to support decision-makers from healthcare policy and insurance companies in determining which therapy options should already be reimbursable by them at this point in time for the indication ME/CFS.

## Einführung: Myalgische Enzephalomyelitis/Chronisches Fatigue-Syndrom

Myalgische Enzephalomyelitis/Chronisches Fatigue-Syndrom (ME/CFS) ist eine schwere, chronische Multisystemerkrankung, die je nach Ausprägung zu erheblichen körperlichen und kognitiven Einschränkungen, zum Verlust der Arbeitsfähigkeit bis hin zur Pflegebedürftigkeit einschließlich künstlicher Ernährung und in sehr schweren Fällen sogar zum Tod führen kann [1–3]. Es handelt sich um eine Erkrankung, die u. a. das zentrale und das autonome Nervensystem, das Immunsystem, das kardiovaskuläre System – insbesondere Endothelzellen der Gefäße – und die Energiegewinnung in den Mitochondrien betrifft, zusätzlich das Darmmikrobiom wie auch die Perfusion von Muskulatur, Gehirn und anderen Organen. Des Weiteren kann es zur Schädigung dünn myelinisierter Aδ-Fasern und/oder unmyelinisierter C‑Fasern und zu einer Reaktivierung von sich latent im Körper befindlichen Viren kommen, wie z. B. Herpesviren, die wiederum weitere Schäden verursachen können [1, 4–6]. Jüngere Studien weisen zudem auf eine Neuroinflammation hin [7–9]. Weil in der Routinediagnostik bisher noch keine spezifischen Biomarker verfügbar sind, wird die Erkrankung nach international konsentierten klinischen Kriterien wie den Kanadischen Konsensuskriterien (CCC) diagnostiziert [1, 2, 10].

ME/CFS ist in ca. 80% der Fälle Folge einer Infektion und tritt in der postakuten Phase auf [1–3, 11]. Zu den dokumentierten Auslösern von ME/CFS zählen neben Infektionen auch chirurgische Eingriffe, Wiederbelebungsmaßnahmen oder Traumata im Bereich des Schädels und der Halswirbelsäule [6, 12]. Bei einem Teil der Betroffenen ist der Auslöser unbekannt. ME/CFS kann auch schleichend oder episodisch beginnen [1–3]. Mindestens zwei Drittel der ME/CFS-Betroffen sind Frauen. ME/CFS kann in allen Altersgruppen auftreten, wobei der Altersgipfel seit der zusätzlichen ME/CFS-Fälle durch die SARS-CoV‑2 Pandemie zwischen 30 und 50 Jahren liegt. ME/CFS kann sich auch schon bei Kindern und Jugendlichen manifestieren. Präpandemisch waren zwei Altersgipfel zu sehen (10–19 Jahre und 30–39 Jahre) [13]. Folglich manifestiert sich ME/CFS hauptsächlich bei Individuen, die sich in der Ausbildung befinden oder aktiv im Berufs- und Familienleben engagiert sind, was eine der leistungsfähigsten Lebensphasen darstellt [1].

Internationale Daten zeigen, dass vor der SARS-CoV-2-Pandemie 0,3–0,9% der Gesamtbevölkerung betroffen waren [14]. Für Deutschland hat das Institut für Qualität und Wirtschaftlichkeit im Gesundheitswesen (IQWiG) präpandemisch 140.000 bis 310.000 Betroffene geschätzt, darunter 70.000–90.000 schulpflichtige Kinder und Jugendliche im Alter zwischen sechs und 17 Jahren [15]. Für Österreich und die Schweiz liegen die Schätzungen je zwischen 26.000 und 80.000 Erwachsene, Kinder und Jugendliche. Ein Großteil der ME/CFS-Betroffenen lebt ohne (korrekte) Diagnose [16]. Seit Jahrzehnten konnten ME/CFS Fälle als Folge von Epidemien und Pandemien dokumentiert und auch in der SARS-Pandemie 2002/03 und der Pandemie durch H1N1 2009/10 beobachtet werden [17, 18]. Studien zufolge ist aufgrund der SARS-CoV-2-Pandemie mindestens mit einer Verdoppelung der Anzahl der Betroffenen zu rechnen [6, 19]. Gemäß der Kassenärztlichen Bundesvereinigung (KBV) in Deutschland wurde ein Anstieg der ME/CFS-Behandlungsfälle von 350.000 bzw. 400.000 in den Jahren 2018 und 2019 auf etwa 500.000 im Jahr 2021 verzeichnet [20]. Für die USA berichteten die Centers for Disease Control and Prevention (CDC) im November 2023, dass im Zeitraum 2021 bis 2022 1,3% der erwachsenen U.S.-Bevölkerung an ME/CFS erkrankt waren, allerdings wurde nur mittels Interview gefragt, ob jemals eine ME/CFS diagnostiziert wurde und jetzt noch besteht [21]. Für Österreich, Deutschland und die Schweiz kann mangels der Verfügbarkeit aktueller Daten keine genaue Anzahl an ME/CFS-Betroffenen genannt werden. Zieht man die internationalen Daten heran, so muss für den D‑A-CH-Raum mit zusätzlich Hunderttausenden Betroffenen, auch Kindern und Jugendlichen, durch die Pandemie gerechnet werden.

Die Symptome von Patient:innen mit ME/CFS im Kontext eines Post-COVID-Syndroms (PCS) waren 20 Monate nach der initialen Infektion im Vergleich zu Patient:innen mit PCS ohne ME/CFS nicht nur schwerer, sondern zeigten auch zumeist keine Besserungstendenz [22].

Die Literatur zur Sterblichkeit im Zusammenhang mit ME/CFS ist spärlich. Verschiedene Studien legen aber eine verkürzte Lebenserwartung für Patient:innen mit ME/CFS durch Herzversagen, Krebserkrankungen (hier vor allem Lymphdrüsenkrebs) und ein erhöhtes Risiko für Suizide nahe [23, 24].

Seit 1969 ist ME/CFS als neurologische Erkrankung von der Weltgesundheitsorganisation (WHO) anerkannt. In der Deutschen Modifikation der zehnten Revision der Internationalen statistischen Klassifikation der Krankheiten und verwandter Gesundheitsprobleme (ICD-10 GM) wird ME/CFS mit dem Code G93.3 verschlüsselt. In den U.S. (ICD-10 CM) wurde der Code 93.32 zur Abgrenzung von anderen Ursachen einer chronischen Fatigue eingeführt. Internationale Institutionen wie die CDC (U.S.) [25] und das National Institute for Health and Care Excellence (NICE) (UK) haben Leitlinien zu ME/CFS erstellt [2]. Zur Diagnostik stehen etablierte klinische Kriterienkataloge zur Verfügung (siehe Kapitel „Diagnostik von ME/CFS“).

Weitere Informationen sowie akkreditierte Fortbildungen finden Sie auf der Seite der Charité Universitätsmedizin Berlin (https://cfc.charite.de/fuer_aerzte/literatur_und_weiterbildung/), der Seite der Deutschen Gesellschaft für ME/CFS (https://www.mecfs.de/) und auf der Seite der Österreichischen Gesellschaft für ME/CFS (https://mecfs.at/aerztinnen/).

## Entstehung und Zielsetzung des D-A-CH-Konsensusstatements

Ausgangspunkt für dieses D‑A-CH-Konsensusstatement zur Diagnostik und Therapie von ME/CFS war die Erarbeitung des Zusatzkapitels „ME/CFS“ für das österreichische Webtool „Management postakuter Zustände am Beispiel Post-COVID-19“, [26] welches auf der gleichnamigen österreichischen S1-Leitlinie basiert, [27] durch eine österreichische Expert:innengruppe. Dieses Ursprungsdokument wird durch das hier vorliegende Konsensusstatement ersetzt werden. Hierzu wurden klinische und wissenschaftliche Expert:innen zum Thema ME/CFS aus dem D‑A-CH-Raum eingeladen, um das Ursprungsdokument weiterzuentwickeln. Die Mehrheit der beteiligten Expert:innen (Ärzt:innen und Therapeut:innen) aus dem D‑A-CH-Raum hat, neben ihrer wissenschaftlichen Expertise, jahre- bis jahrzehntelange klinische Erfahrung in der Betreuung und Behandlung von ME/CFS-Patient:innen. Darüber hinaus war es allen Beteiligten besonders wichtig, auch ME/CFS-Patient:innen-Organisationen aus allen drei Ländern auf Augenhöhe im Sinne einer State-of-the-art-Patient:innen-Beteiligung in den Konsensus-Prozess mit einzubinden.

Das Ausgangsdokument wurde von allen Autor:innen in fünf Überarbeitungsrunden für den D‑A-CH-Raum erweitert, aktualisiert und optimiert. Dieses Konsensusstatement fokussiert bei der Diagnostik auf die Kanadischen Konsensuskriterien (CCC) für ME/CFS. Diese sind spezifischer als z. B. die klinischen Kriterien des Institute of Medicine (IOM) für ME/CFS und sowohl in der Forschung als auch in der klinischen Praxis anerkannt.

Für die Diagnostik in der Kinder- und Jugendheilkunde könnten die CCC jedoch teilweise zu stringent sein. Aus diesem Grund wird angestrebt in absehbarer Zeit für die Kinder- und Jugendheilkunde ein zusätzliches, über dieses Konsensusdokument hinausgehendes Statement zu verfassen. Im hier vorliegenden Basisdokument wird der Bezug auf die CCC jedoch für alle Altersgruppen belassen.

ME/CFS-Kriterien, welche das Leitsymptom von ME/CFS, die post-exertionelle Malaise (PEM), nicht berücksichtigen und/oder mit einer pathologischen Fatigue vermischen, sind veraltet und wurden daher ausgeschlossen.

Grundsätzlich ist dieses ME/CFS-Konsensusstatement nicht als abgeschlossen anzusehen, da fortlaufend neue wissenschaftliche Erkenntnisse zu ME/CFS gewonnen und publiziert werden.

Das Ziel dieses D‑A-CH-Konsensusstatements ist es,

- den aktuellen Wissensstand zu ME/CFS zusammenzufassen,
- in der Diagnostik die CCC als klinische Kriterien mit Fokus auf das Leitsymptom post-exertionelle Malaise (PEM) hervorzuheben und
- vor allem im Hinblick auf Diagnostik und Therapie einen Überblick über aktuelle Optionen und mögliche zukünftige Entwicklungen aufzuzeigen.

Das D‑A-CH-Konsensusstatement soll Ärzt:innen, Therapeut:innen und Gutachter:innen dabei unterstützen, Patient:innen mit Verdacht auf ME/CFS mittels adäquater Anamnese und klinisch-physikalischen Untersuchungen sowie der empfohlenen klinischen CCC zu diagnostizieren und dabei die in Tab. 1 präsentierten Fragebögen sowie weiteren Untersuchungsmethoden zu nutzen. Der Überblick über die zwei Säulen der Therapie bei ME/CFS, Pacing und die symptomlindernden Therapieoptionen (zusammengefasst in Box 1) sollen nicht nur Ärzt:innen und Therapeut:innen zur Orientierung dienen, sondern auch Entscheidungsträger:innen aus der Gesundheitspolitik und den Versicherungen darin unterstützen, welche Therapieoptionen bereits zu diesem Zeitpunkt bei der Indikation „ME/CFS“ von diesen erstattbar sein sollten.

**Tab. 1:** Überblick über die Kanadischen Konsensuskriterien, Haupt- und Nebenkriterien, mögliche entsprechende Symptome, unterstützende Fragebögen und Untersuchungsmethoden und -werkzeuge, die im Rahmen von Studien auf eine verbesserte Diagnostik hindeuten

Kanadische Konsensuskriterien	Mögliche entsprechende Symptome	Unterstützende Fragebögen	Differenzialdiagnostik und Untersuchungsmethoden, die in Studien auf eine verbesserte Diagnostik hindeuten
Post-exertionelle Malaise (PEM)	Belastungsintoleranz, Symptomexazerbation, Zustandsverschlechterung nach Überlastung, physiologische Aktivitäts-Erholungs-Dysfunktion Diese Verschlechterung tritt meist zeitverzögert (12–72h) auf und dauert in der Regel mind. 14–24h an, kann aber auch Tage oder länger anhalten. Auch eine permanente Verschlechterung ist möglich. Für weitere Information siehe auch: https://www.mecfs.de/was-ist-me-cfs/pem/	DePaul-Symptom Questionnaire für PEM^a^ (DSQ-PEM) [76] Munich Berlin Symptome Questionnaire^b^ (MBSQ) [75], Teil II	**a)** *Abklärung von pathologischen Befunden in Ruhe (eingeschränkte Sauerstoffversorgung der Muskulatur, gestörter Energiestoffwechsel, Amyloidablagerungen, Mikrogefäßschäden, mitochondriale Dysfunktion, arterieller Gefäßsteifigkeit) (in Studien*): [38, 40, 42, 77]
			Angiologische Untersuchungen mittels EndoPAT bzw. flussvermittelter Vasodilatation (FDM) an der A. brachialis oder mittels postocclusiver-reaktiver Hyperämie (PORH) – Messung, Oxygen-To-See (O2C), Nagelfalzmikroskopie, venöses Pooling
			Analyse der retinalen Mikrozirkulation [78]
			Blutuntersuchung auf zirkulierende Mikroclots [79–82]
			Blutuntersuchung oder Muskelbiopsie zur Abklärung einer mitochondrialen Dysfunktion [1, 83–86]
			Blutuntersuchung auf erhöhte Laktatwerte [87–89]
			**b)** *Abklärung einer pathologische physiologische Aktivitäts-Erholungsreaktion*:
			Doppelte Handkraftmessung [41] mit jeweils 10 Zügen im Abstand von mindestens einer Stunde
			Kardiopulmonaler Belastungstest an zwei aufeinander folgenden Tagen im Abstand von 24 Stunden: [42, 44] Da dieser Test bei ME/CFS-Erkrankten zu einer dauerhaften Verschlechterung des Gesundheitszustandes führen kann, soll das Verfahren außerhalb von Studien nicht [39, 43] und auch dann nur wie von Faghy et al. 2024 beschrieben, adaptiert angewendet werden [90]
			Blutuntersuchung auf inadäquate Laktatakkumulation [87–89]
Pathologische Fatigue mit Einschränkungen der Alltagsfunktion	„Zu den vorausgegangenen Anstrengungen unverhältnismäßige, durch Schlaf nicht zu beseitigende körperliche, geistige und/oder seelische Erschöpfung“ [12]	Fatigue Assessment Scale^c^ (FAS) Fatigue Skala (FS) [91] Fatigue Severity Scale (FSS) [92] Chalder Fatigue Questionnaire (CFQ) [93] MBSQ [75], Teil I	Differentialdiagnostik/Abgrenzung anderer Ursachen für Fatigue, falls entsprechende klinische Hinweise vorliegen, [6] z. B. Sarkoidose, Lupus erythematodes (LE), Morbus Bechterew, Psoriasisarthritis, Sjögren-Syndrom, Kollagenosen, Fibromyalgie, Diabetes mellitus, Hypothyreose, Hashimoto-Thyreoiditis, Morbus Addison, Hyperkalzämie, Endometriose, Anämie, Eisenmangel, Vitaminmangel, maligne Erkrankungen („Tumorfatigue“), Z. n. onkologischer Therapie, angeborene Immundefekte (z. B. CVID), postinfektiöse Fatigue ohne ME/CFS, posturales Tachykardiesyndrom (POTS) und andere Dysautonomie-Syndrome, ZNS-Borreliose, AIDS, chronische Sinusitis, chronische Hepatitis, Mastzellaktivierungssyndrom (MCAS), Depression
			*Anmerkung*: [6] Es ist entscheidend, stets nach dem Vorliegen von PEM zu fragen, um ME/CFS von anderen Erkrankungen mit Fatigue zu unterscheiden (z. B. Fibromyalgie, Depression). Jedoch schließen Komorbiditäten wie z.B. Hashimoto-Thyreoditis, Fibromyalgie, POTS oder ein hypermobiles Ehlers-Danlos-Syndrom eine ME/CFS auch nicht aus
Schlafstörungen	Einschlafstörung, Durchschlafstörung, Verschobener Schlaf‑/Wachrhythmus, Nicht erholsamer Schlaf	Jenkins Sleeping Scale [94] (kurz) MBSQ [75], Teil III	*Abklärung anderer Ursachen von Schlafstörungen*:
			Overnight Oxymetrie zu Hause [95]
			Screening auf obstruktive Schlafapnoe (OSAS) bei V. a. Monotonie-Intoleranz
			Schlaflabordiagnostik bei sehr ungewöhnlichen Schlafmustern oder Verdacht auf obstruktive Schlafapnoe [6]
Schmerzen	Kopfschmerzen, Muskel‑/Gelenkschmerzen, Nervenschmerzen, Knochenschmerzen, Parästhesien, Temperaturstörungen, Sensibilitätsstörungen, „pins and needles“, Muskelzucken, „restless legs“, Hypermobilität [96]	MBSQ [75], Teil IV Somatic Symptom Scale – 8 (SSS-8) [97] SFN-SIQ Fragebogen zur Einschätzung einer Small-Fiber-Neuropathie [98]	**a)** *Differentialdiagnostik/Abgrenzung anderer Ursachen, v.* *a. Migräne, HWS-Syndrom, Polyneuropathie (PNP), ZNS-Borreliose, Durchführung neuroorthopädischer Status* [99]
			**b)** *Abklärung einer Small-Fiber-Neuropathie (SFN)*
			Hauptbiopsie und neuropathologische Untersuchung in einem spezialisierten Labor (PGP 9.5), ev. zusätzlich mit Beurteilung der Mastzellen [50, 100]
			Testung der Schweißsekretion oder der elektrischen Leitfähigkeit der Haut, z. B. mittels Sudoscan oder Biofeedback-Untersuchung
			**c)** *(Re‑)Infektionsdiagnostik und -Labor, v.* *a. bei chronischen Kopfschmerzen*
			**d)** *Abklärung einer Hypermobilität*: [101]
			Beighton-Score, Checkliste für hypermobiles Ehlers-Danlos-Syndrom (hEDS) [102]
			evtl. Zeichen einer kraniozervikalen Instabilität (CCI) [103]
			**e)** *Abklärung des Tethered-Cord-Syndroms (TCS)*:
			Sakrale Grübchen/Haaren, evtl. LWS-MRT in Bauchlage (wenn verfügbar) [104]
			**f)** *Abklärung eines POTS (mögliche Erklärung für Kopf- und Nackenschmerzen) (siehe auch „autonome Manifestationen“)* [102, 105]
			**g) ***Abklärung von Mastzellüberaktivität/MCAS als mögliche Ursache für Kopf- und andere Schmerzen* [102, 106]
Neurologische/kognitive Manifestationen	Kurzzeitgedächtnis-, Konzentrationsstörungen, Aufmerksamkeitsstörungen, „brain fog“, Wortfindungsstörungen, Reizempfindlichkeit (Licht, Geräusche Berührungen), Ataxien, Muskelzuckungen, Muskelschwäche	MBSQ [75], Teil V MoCA-Test, [107] Reaktionszeit-Test [108]	**a)** *Differentialdiagnostik/Abgrenzung anderer Ursachen von kognitiven Defiziten*: [99] z. B. MS, Demenz
			cMRT, (Re‑)Infektionsdiagnostik, neurologische/psychiatrische Diagnostik
			**b)** *Abgrenzung von sekundären, reaktiven psychischen Problemen/Störungen (z.* *B. Depression, Ängste): Siehe dazu Grande et al. 2023 [*71*] und Gole 2023* [109]
			**c)** *Neuropsychologische Untersuchung mit Fokus auf ME/CFS-typische Probleme bzgl. Konzentration, Aufmerksamkeit, Kurzzeitgedächtnis, verlangsamte Informationsverarbeitung* [110–112]
			**Cave**: Auch hier ist es wichtig, PEM zu berücksichtigen und den MoCA-/Reaktionszeit-Test im Abstand von einer Stunde wiederholt durchzuführen
			**d)** *Screening auf Aufmerksamkeitsdefizitsyndrom (ADHS)*, [113] *v.* *a. im Hinblick auf die Adhärenz in Bezug auf Medikamenteneinnahme und Pacing*
			**e)** *Abklärung eines POTS, da dabei Probleme mit Konzentration, Aufmerksamkeit, Kurzzeitgedächtnis, verlangsamter Informationsverarbeitung häufig sind*: [112]
			Schellong-Test
			NASA-Lean-Test^d^
			Kipptischuntersuchung (mit Near-Infrared Spectroscopy oder Carotis – Sono um Durchblutungsstörungen zu erkennen) [45, 46]
			**f)** *Abklärung von Mastzellüberaktivität/MCAS als mögliche Ursache für kognitive Einschränkungen*
			**g)** *Blutuntersuchung auf zirkulierende Mikroclots (kognitive Einschränkungen durch z.* *B. eingeschränkte Sauerstoffversorgung) (Studien)* [79–82]
			**h) ***Bildgebung zur Feststellung von metabolischen oder Perfusionsstörungen, z.* *B. PET-MRT (nur in Studien)*
Autonome Manifestationen, v. a. orthostatische, aber auch vasomotorische, sekreto-/sudomotorische, gastrointestinale, urogenitale, pupillomotorische Dysfunktionen	Palpitationen, Tachykardie, Schwindel, Ohnmachtsneigung, kognitive Dysfunktionen, Kurzatmigkeit, vermehrtes Schwitzen, Temperaturregulationsstörungen, Schluckstörung, Verdauungsstörungen, Sehstörung, Lichtempfindlichkeit, Harnblasendysfunktionen	COMPASS31-Fragebogen^e^ [114] MBSQ, [75] Teil VI SFN-SIQ [98]	**a)** *Differentialdiagnostik/Abgrenzung anderer Ursachen siehe auch Frontera et al. 2023* [99] *und Blitshteyn et al. 2022* [115]
			**b) ***Abklärung von POTS und OH*:
			Schellong-Test
			NASA-Lean-Test^d^
			Kipptischuntersuchung (mit Near-Infrared Spectroscopy oder Carotis-Sono um Durchblutungsstörungen zu erkennen) [45, 46]
			Bei OH auch Abklärung einer Nebenniereninsuffizienz
			**c) ***Abklärung von Mastzellüberaktivität/MCAS als mögliche Ursache von Palpitationen, Tachykardie, Schwindel, vermehrtem Schwitzen, Verdauungsstörungen etc.*
			**d) ***Abklärung einer SFN als Ursache von sekreto-/sudomotorischer Dysfunktion*
			Hauptbiopsie und neuropathologische Untersuchung [50, 100]
			Testung der Schweißsekretion oder der elektrischen Leitfähigkeit der Haut, z. B. durch Sudoscan oder Biofeedback
Neuroendokrine Manifestationen	Durstregulationsstörungen, Blutdruckstörungen, Schwitzen, Störungen des Tag-Wachrhythmus, Temperaturregulationsstörungen, Appetitlosigkeit, Schilddrüsenfunktionsstörungen, Nebennierenstörungen, Insulinresistenz	MBSQ, [75] Teil VII	**a)** *Differentialdiagnostik/Abgrenzung anderer Ursachen*
			**b)** *Abklärung von Laborauffälligkeiten, die mit ME/CFS einhergehen können* [116, 117]
			Thyreotrope Achse (HPT): z. B. niedrige Schilddrüsenhormonaktivität [118]
			Somatotrope Achse (HPS): z. B. mangelhafte Regulierung des Wachstumshormons (GH), [119] was zu Muskel- und Knochenschwund, Muskelschwäche, Glukose- und Fettstoffwechselstörung führen kann
			Gonadotrope (HPG)-Achse: evtl. frühere Menopause [120], Immundysregulation [121]
			Störungen der adrenokortikalen (HPA) Achse sollte niedrigschwellig getestet werden durch z.B.: Synacten-Test, Cortisol-Tagesprofil im Speichel, Osmolalität im Harn und Blut [116, 122, 123]
			**c)** *Insulinresistenz (HOMA-Index)* [124, 125]
			**d)** *Neurotransmitteranalyse im Harn*: [126] evtl. verminderte Katecholamine, Aminosäuren
			**e) ***MRT-Hypophyse bei anamnestischen/laborparametrischen Hinweisen*
Immunologische Manifestationen	Symptome, die einer Mastzellüberaktivität/einem diagnostizierten Mastzellaktivierungssyndroms (MCAS) entsprechen wie kardiale Symptome (Palpitation, Tachykardie, Synkopen), kutane Symptome (Urtikaria, Pruritus, Flush und Angioödem), respiratorische Symptome (Asthma, erschwertes Atmen, nasale Kongestion) und gastrointestinale Symptome (Schmerzen, Durchfall, Übelkeit und Erbrechen) [127–130]. Immunologische Dysfunktionen wie deutlich häufigere und/oder schwere Infektionen	MBSQ, [75] Teil VIII	**a)** *Differentialdiagnostik/Abgrenzung anderer Ursachen wie z.* *B. Allergien, Asthma, Nahrungsunverträglichkeiten, chronisch-entzündliche Darmeerkrankungen, Zöliakie, Small Intestinal Bacterial Overgrowth (SIBO), Leaky-Gut-Syndrom bei gastrointestinalen Symptomen bzw. POTS und kardiale/pulmologische Ursachen bei den kardialen/pulmologischen Symptomen*
			**b)** *MCAS-Diagnostik laut Konsensus* [127–130] *(Messung von Tryptase, Histamin und DAO-Aktivität im Blut unter Beachtung der entsprechenden Präanalytik sowie von Histamin-Abbauprodukten und Leukotrienen im Urin). Histamin und DAO-Aktivität hauptsächlich zur Abgrenzung einer Histaminintoleranz*
			*Anmerkung:* Bei den meisten ME/CFS-Patient:innen liegt keine MCAS, sondern eine Mastzellüberaktivität vor [131]
			**c)** *Abklärung humoraler/zellulärer Immundefekte bei pathologischer Infektionsanfälligkeit entsprechend des Erregerprofils*
			**d)** *Infektionsdiagnostik bei spezifischen Infekt-assoziierten Symptomen*
			**e) ***Abklärung weiterer Autoimmun- bzw. rheumatologischer Erkrankungen*: [6]
			z. B. Hashimoto-Thyreoiditis, bei ANA-Erhöhung Screening auf Antikörper gegen extrahierbare nukleäre Antigene (ENA) und auf Anti-dsDNS-Antikörper [132]
			Bei Sicca-Symptomatik Sjögren-Syndrom ausschließen [6]
			**f)** *In Studien: Autoantikörper gegen adrenerge, muskarinerge und andere G‑Protein-gekoppelte Rezeptoren (GPCR); sie lassen sich bei einem Teil der Patient:innen nachweisen, sind aber nicht erkrankungsspezifisch*
			**g)** *In Studien: Dysbioseanalyse*
			**h)** *In Studien: Immuno-PET* [133]

^a^ https://csh.depaul.edu/about/centers-and-institutes/ccr/myalgic-encephalomyelitis-cfs/Pages/measures.aspx

^b^ https://static-content.springer.com/esm/art%3A10.1007%2Fs00431-023-05351-z/MediaObjects/431_2023_5351_MOESM2_ESM.pdf

^c^ https://oegam.at/system/files/abb5.pdf

^d^ https://batemanhornecenter.org/nasa-10-minute-lean-test-2/, https://www.pots-dysautonomia.net/_files/ugd/9808d3_a4c80550d5e54fdf8251e9a89e15717f.pdf

^e^ https://bmjopen.bmj.com/content/bmjopen/11/1/e038677/DC3/embed/inline-supplementary-material-3.pdf?download=true

## Diagnostik von ME/CFS

Eine der großen Herausforderungen für ME/CFS-Erkrankte ist es, die korrekte Diagnose zu bekommen [28, 29]. Internationale Studien gehen davon aus, dass 84–90% der ME/CFS-Betroffenen nicht oder fehldiagnostiziert sind [16]. In Deutschland vergehen im Schnitt sechs bis sieben Jahre bis zur Diagnosestellung [30]. In Österreich wurde erhoben, dass zwischen dem Beginn der Symptome und der Diagnosestellung durchschnittlich fünf bis acht Jahre vergehen, wobei bei 30% der Betroffenen die Zeitspanne sogar über zehn Jahre hinausging [31, 32]. In der Schweiz beträgt diese Dauer im Durchschnitt knapp sieben Jahre und die Patient:innen mussten mehr als elf unterschiedliche Ärzt:innen aufsuchen, bis nach durchschnittlich 2,6 Fehldiagnosen die korrekte Diagnose ME/CFS gestellt wurde [33].

In dieser Zeitspanne kann sich der Gesundheitszustand der Betroffenen unumkehrbar verschlechtern. Allerdings kann eine frühzeitige und korrekte Diagnosestellung von ME/CFS sowie eine fundierte Aufklärung die Aussichten für die Erkrankten verbessern [34].

Problematisch für den niedergelassenen Bereich bleibt, dass es weder in Österreich noch in der Schweiz eine Spezialambulanz zur Überweisung von Patient:innen gibt, bei denen sich die Verdachtsdiagnose ME/CFS anhand des klinischen Bildes und einer umfassenden Differentialdiagnostik bestätigt hat. In Deutschland besteht für Erwachsene aus dem Einzugsgebiet Berlin/Brandenburg die Möglichkeit, eine einmalige Diagnostik einschließlich eines Therapieplans in der Immundefekt-Ambulanz der Charité – Universitätsmedizin Berlin zu erhalten. Eine Versorgung von Patient:innen bis zu einem Alter von 20 Jahren bietet das auf ME/CFS spezialisierte MRI Chronische Fatigue Centrum für junge Menschen (MCFC) der Technischen Universität München (TUM) und München Klinik gGmbH an. Aus Kapazitätsgründen liegt der Fokus hier auf jungen Betroffenen aus Bayern.

Im Rahmen dieses Kapitels werden grundlegende und weiterführende Möglichkeiten zur Diagnostik als Orientierung aufgezeigt. Diese können die strukturelle Lücke jedoch bei weitem nicht schließen.

ME/CFS wird häufig mit einer psychischen Krankheit verwechselt. Auch nimmt ein Teil der Ärzt:innen und Therapeut:innen fälschlicherweise psychische bzw. psychosomatische Faktoren als ursächlich für die Entstehung und Chronifizierung von ME/CFS an [35–37]. Mit moderner Diagnostik lassen sich allerdings pathobiologische Dysfunktionen und organpathologische Befunde nachweisen, wie etwa Einschränkungen der kardiopulmonalen Leistungsfähigkeit bei wiederholter Belastung [38–44] sowie Einschränkungen der Gehirndurchblutung [45]. Während etwa bei Kipptischanalysen die Durchblutung des Gehirns von gesunden Proband:innen im Schnitt nur um 7% abfällt, sinkt sie bei Patient:innen mit ME/CFS um durchschnittlich 26% [6, 46]. Histologisch und elektronenmikroskopisch zeigen sich eine Schädigung der Mitochondrien, Rarefizierung der Kapillaren und Nekrosen der Muskulatur nach Belastung [38]. Auch im Immunprofil finden sich Auffälligkeiten [47–50].

Bislang wurde jedoch noch kein spezifischer und praxistauglicher Biomarker für die Erkrankung identifiziert. Dies könnte auch daran liegen, dass die Erkrankten in den Studien oftmals nicht ausreichend charakterisiert und stratifiziert wurden. Eine Studie aus Österreich zeigte z. B., dass sich bei Menschen mit ME/CFS und Immundefekten andere potenzielle Marker nachweisen ließen als bei Betroffenen ohne zusätzlichen Immundefekt [51]. Bei Patient:innen mit PCS und ME/CFS zeigte sich, dass auch Frauen und Männer unterschiedliche Marker aufweisen [52, 53]. Zudem könnte hier auch die Dauer der Erkrankung sowie der aktuelle Gesundheitszustand der Patient:innen (beispielsweise Gesundheitszustand ohne akute Exazerbation durch post-exertionelle Malaise (PEM) und während der PEM (siehe unten)) eine Rolle spielen, wie Xiong et al. 2023 aufzeigten [54].

Daher muss die Diagnose durch klinische Kriterien, klinische Untersuchungen und durch die Abgrenzung zu anderen Erkrankungen gestellt werden. Es handelt sich dabei um ein Verfahren, mit dem Ärzt:innen und insbesondere auch Neurolog:innen bereits von anderen Krankheiten her vertraut sind, wie etwa der Migräne, dem postkommotionellen Syndrom, dem Restless-Leg-Syndrom oder auch der Alzheimer-Demenz.

**In der Abgrenzung von anderen Erkrankungen kommt dem Leitsymptom von ME/CFS, der post-exertionellen Malaise (PEM), eine entscheidende Bedeutung zu. Sie ist für die Diagnosestellung zwingend erforderlich** [6].

PEM ist eine belastungsinduzierte, unverhältnismäßige Zustandsverschlechterung durch eine gestörte physiologische Aktivitäts-Erholungsreaktion [38, 42, 43, 55]. Die Verschlechterung und/oder das Aufkommen neuer Symptome (sog. „Crash“) treten unmittelbar oder oft zeitverzögert (12–72 Stunden) nach bereits geringer physischer, kognitiver, mentaler, orthostatischer oder sensorischer Belastung auf, die vormals toleriert wurde [56]. Die Verschlechterung kann Stunden, Tage oder gar Wochen anhalten (unterschiedliche Übersichtsarbeiten sprechen von mind. 14–24 Stunden). Jeder „Crash“ birgt das potenzielle Risiko einer permanenten Verschlechterung des Gesamtzustandes.

Durch das Leitsymptom der PEM lässt sich ME/CFS von anderen Krankheiten, die von Fatigue begleitet sind, am besten abgrenzen, beispielsweise von der Multiplen Sklerose (MS) [57]. Eine besonders sorgfältige Abgrenzung zu psychischen Erkrankungen wie z. B. Depression oder Burn-out, ist von größter Wichtigkeit, um Fehldiagnosen und Fehlbehandlungen zu vermeiden [6, 58]. Auch wenn bei beiden, ME/CFS und Depression die Symptome Fatigue und Schlafstörungen bestehen, **unterscheidet PEM die Krankheiten deutlich**: [6] Während Patient:innen mit Depression oder Burn-out unter einer ausgeprägten Motivations- und Antriebsstörung leiden, sind Motivation und Antrieb bei ME/CFS-Betroffenen unvermindert. Für letztere führt eine Überlastung (z. B. durch Sport oder aktivierende Rehabilitationsmaßnahmen) zu einer Verschlechterung des Zustandes, während Patient:innen mit Depression oder Burn-out von aktivierenden Maßnahmen in der Regel profitieren. Für ME/CFS-Betroffene steht daher „Pacing“ statt Aktivierung im Zentrum des Krankheitsmanagements.

Weitere häufige Fehldiagnosen sind die somatoformen Störungen, die sich durch eine sorgfältige interdisziplinäre Differenzialdiagnostik abgrenzen lassen. Auch hier ist das Leitsymptom PEM bei der Differenzierung entscheidend. Die Diagnose einer somatoformen Störung zielt darauf ab, dass die von den Patient:innen präsentierte Symptomatik und die damit verbundenen Einschränkungen nicht hinreichend durch somatische Befunde erklärt werden können und dass ihr Krankheitserleben und -verhalten insofern als auffällig bzw. dysfunktional beurteilen wird. Es ist offensichtlich, dass diese Diagnose statt der Diagnose ME/CFS nur dann möglich ist, wenn Existenz und Bedeutung der inzwischen vorliegenden pathobiologischen und organpathologischen Substrate nicht gekannt oder ignoriert werden [59, 60]. Insofern ist auch die mit der Diagnose behauptete Kausalrichtung in der Weise umzukehren, dass Krankheitserleben und -verhalten von ME/CFS-Erkrankten reaktiv auf die körperlichen Symptome, die von den ME/CFS-Erkrankten überwiegend als sehr schlecht bezeichnete Arzt-Patient-Beziehung und nicht zuletzt auch auf die inadäquate Versorgung folgen [30, 61–65]. Den Krankheitsverlauf moderierende psychische Einflussfaktoren können grundsätzlich vorhanden sein, sind aber auch bei Betroffenen anderer schwerer körperlicher Krankheiten anzutreffen und deshalb höchst unspezifisch. Die Fehldiagnose „Neurasthenie“ wurde zu Beginn des 20. Jahrhunderts häufig vergeben, [66] aber von der WHO im Zuge der ICD-11 als überholt eingestuft [67].

Sekundär können sich – vor allem durch die fehlende und/oder falsche medizinische Versorgung, Stigmatisierung, soziale Isolation und fehlende soziale Absicherung – bei ME/CFS-Betroffenen Traumatisierungen oder eine depressive Reaktion bis hin zu erhöhter Suizidalität entwickeln [68–70]. ME/CFS-Patient:innen sollten je nach Schweregrad der Erkrankung bestmöglich symptomorientiert behandelt werden. Dabei müssen alle Maßnahmen an die PEM und damit verbundene Risiken angepasst werden. Dies gilt auch für diesbezügliche Komorbiditäten. Grundsätzlich gilt hier der Grundsatz „Pacing first“ (s. unten), der dem Einsatz mobilisierender Techniken in Psychotherapie und Rehabilitation vorgeordnet ist. Zudem müssen die Dosierungen der Medikamente ebenso wie der Zeitpunkt der Medikamenteneinnahme auf ME/CFS abgestimmt werden. ME/CFS-Betroffene reagieren zum Teil bereits auf geringe Dosierungen [6, 71].

Ähnlich wie bei der MS und vielen anderen Autoimmunerkrankungen kann der erste Eindruck zum Allgemeinzustand über die Schwere der Erkrankung und den Grad der Einschränkungen im Alltag hinwegtäuschen [6]. Im Rahmen der körperlichen Untersuchung zeigt sich oft, dass die Hände kalt und möglicherweise feucht sind, begleitet von einer leichten Blaufärbung der Extremitäten oder Raynaud-Phänomenen sowie einer marmorierten Beschaffenheit der Haut. Häufig ist der Puls in Ruhephasen erhöht. Des Weiteren treten oft gerötete Augen auf. Bei schwer betroffenen Personen oder während Phasen der Verschlechterung kann das Gesicht geschwollen erscheinen [6]. Die Patient:innen können auch Lymphknotenschwellungen und Halsschmerzen haben, die mit entzündlichen Veränderungen der Mundschleimhaut einhergehen [28].

**Daher ist es zwingend zur klinischen Diagnostik auch die Einschätzung des Schweregrads von ME/CFS adäquat durchzuführen.**

### Klinische Kriterien, Fragebögen, spezifische Untersuchungsmethoden

In den letzten Jahren haben sich neben den weitgefassten IOM-Kriterien, [72] die für eine erste Verdachtsdiagnose geeignet sind, nicht jedoch für die erweiterte Diagnostik und die Forschung, die Kanadischen Konsensuskriterien (CCC) für ME/CFS als klinischer Kriterienkatalog bewährt [1, 10, 73, 74]. Diese stringenten klinischen CCC-Kriterien fordern die Erfüllung von allen fünf Hauptkriterien, zusätzlich die Erfüllung von zwei der drei Nebenkriterien und eine Erkrankungsdauer von mindestens 6 Monaten bei Erwachsenen und 3 Monaten bei Kindern und Jugendlichen.

Hauptkriterien gemäß der CCC:

1. Post-exertionelle Malaise (PEM)
2. Pathologische Fatigue mit Einschränkungen der Alltagsfunktion
3. Schlafstörungen
4. Schmerzen
5. Neurologische/kognitive Manifestationen

Nebenkriterien gemäß der CCC:

- Manifestationen des autonomen Nervensystems
- Neuroendokrine Manifestationen
- Immunologische Manifestationen

Die folgende Tab. 1 gibt einen Überblick über die CCC, deren Haupt- und Nebenkriterien, mögliche den Haupt- und Nebenkriterien entsprechende Symptome, anzuwendende Fragebögen und weitere Untersuchungsmethoden und Werkzeuge, die im Rahmen von Studien zu einer verbesserten Diagnostik beitragen können.

Die in der Tabelle aufgezählten Symptome und Untersuchungsmethoden haben keinen Anspruch auf Vollständigkeit, da durch internationale Forschung fortlaufend neue Erkenntnisse gewonnen werden. Die Laborparameter werden nach Tab. 1 noch einmal zusammenfassend erwähnt. Eine genaue Anamnese inkl. Krankheitshistorie sowie eine genaue klinisch-physikalische Untersuchung entsprechend der Symptome werden vorausgesetzt. Der Munich-Berlin-Symptom-Questionnaire (MBSQ) sollte grundsätzlich als Basis-Erhebungsinstrument verwendet werden, da er auch die Schwere und Häufigkeit der einzelnen Symptome berücksichtigt [75].

Auch wenn Routinelaboruntersuchungen bei ME/CFS meist keine oder wenige Auffälligkeiten zeigen, sollten sie in angemessenem Umfang durchgeführt werden, um alternative Diagnosen oder Komorbiditäten festzustellen.

Darüber hinaus umfasst laut publizierter Übersichtsarbeiten ein sinnvolles Basisscreening für ME/CFS ein Blutbild mit Leukozytendifferenzierung, C‑reaktives Protein, Ferritin, Hämoglobin A_1c_, Kreatinin, Leberwerte, LDH, Bilirubin, Elektrolyte mit Phosphat, thyreoideastimulierendes Hormon inkl. fT3 und fT4, die Immunglobuline G, A und M, antinukleäre Antikörper (ANA), anti-Thyreoperoxidase-Antikörper, Zöliakie-Antikörper und N‑terminales natriuretisches Propeptid vom B‑Typ (NT-proBNP) [6, 28, 134]. Weitere Laboranalysen können abhängig von Auffälligkeiten im Basislabor, anamnestischen, körperlichen Befunden oder im Rahmen der CCC festgestellt, sinnvoll sein (siehe Tab. 1; [6, 28, 134]).

Im Falle einer pathologischen Infektionsanfälligkeit (wiederkehrende Herpesvirusreaktivierungen, Nasennebenhöhleninfektionen, Harnwegsinfekte, Bronchitis, Lungenentzündung und Otitis media) oder klinischer Immundysregulation (Halsschmerzen, Lymphknotenschmerzen, Kopfschmerzen) können serologische (z. B. Herpesvirus-Serologie) und immunologische Analysen (z. B. IgG-Subklassen, Impftiter, zellulärer Immunstatus) wegweisend sein. Bei peripheren Durchblutungsstörungen mit Hinweisen auf eine endotheliale Dysfunktion oder Endotheliitis kann die Analyse von Antikörpern gegen Zytoplasma neutrophiler Granulozyten (ANCA), Cardiolipin, Prothrombin und/oder Angiotensin Converting Enzyme 2 (ACE-2) sinnvoll sein [135]. Ferner sollte eine Analyse des Lipdstoffwechsels (LDL, HDL, VLDL und Gesamtcholesterin), des Zuckerstoffwechsels inkl. HbA1c und Protein C sowie prothrombotischer Faktoren wie Lp(a) und Homocystein umgesetzt werden. Bei Muskelschmerzen und proximaler Muskelschwäche sollte stets auch die Kreatinkinase (CK) zum Ausschluss spezifischer Myopathien bestimmt werden. Bei V. a. neuroendokrine Manifestationen muss auch die thyreotrope, somatotrope, gonadotrope und adrenocorticale Achse untersucht werden. Eine genaue Anamnese zum Ausschluss hepatischer Funktionsstörungen im Sinne einer nichtalkoholischen Steatohepatits ist empfehlenswert, vor allem im Vorfeld einer differenzierten Medikamententherapie mit „off-label“ Medikamenten.

Häufig lassen sich bei ME/CFS-Patient:innen erhöhte Autoantikörper gegen diverse Rezeptoren z. B. der adrenergen und cholinergen Reizübertragung nachweisen, ihre Relevanz für die Therapieplanung ist allerdings noch unklar, so dass ihre Bestimmung, wie auch die Bestimmung von zirkulierenden Mikrothromben, bislang nur im Rahmen von Studien im Hinblick auf künftige Therapieoptionen sinnvoll ist.

Zusätzlich sollte ein besonderes Augenmerk auf eine mögliche orthostatische Dysfunktion des autonomen Nervensystems gelegt werden (Tab. 1). Die orthostatische Intoleranz (OI) ist ein wichtiges Nebenkriterium der CCC und tritt geschätzt bei mindestens 2/3 der ME/CFS-Patient:innen auf [134, 136, 137]. Die OI manifestiert sich häufig als posturales (orthostatisches) Tachykardiesyndrom (POTS), seltener als orthostatische Hypotonie (OH). Zu den orthostatischen Symptomen zählen Schwindel, Benommenheit, Schwäche, Übelkeit, Herzrasen und/oder Ohnmachtsanfälle. Eine orthostatische Belastung kann bei ME/CFS zu einem Crash führen. Wenn der Verdacht auf OI über 3 Monate besteht, sollte je nach Belastbarkeit, ein passiver 10-Minuten-Steh-Test (NASA-Lean-Test) durchgeführt werden, bei dem die Betroffenen im Gegensatz zum Schellong-Test mit den Schultern an eine Wand gelehnt stehen. Empfohlen wird eine minütliche Messung von Puls und Blutdruck. Ein POTS liegt vor, wenn über drei Monate eine OI besteht und im Stehtest ein anhaltender Pulsanstieg nach Aufrichten von mindestens 30 (40 bei Menschen mit einem Alter < 20 Jahre) Schlägen pro Minute festgestellt wird, der von Symptomen begleitet wird, die sich im Liegen wieder bessern. Bei Kindern und Jugendlichen zählt auch ein anhaltender Pulsanstieg im Stehen von absolut mehr als 120 Schlägen pro Minute als POTS. Die OH wird bei einem anhaltenden Blutdruckabfall von mehr als 20 mm Hg oder unter 90 mm Hg systolisch oder mehr als 10 mm Hg diastolisch diagnostiziert, wobei zwischen einer klassischen OH in den ersten drei Minuten und einer verzögerten OH ab Minute 4 unterschieden wird (siehe auch: https://www.pots-dysautonomia.net/_files/ugd/9808d3_a4c80550d5e54fdf8251e9a89e15717f.pdf). Aktuelle Studien mit near-infrared-Spektrographie oder Doppler-Sonographie während einer Kipptischuntersuchung konnten bei einer OI Perfusionsstörungen im Gehirn nachweisen, die bis zu mehrere Stunden anhalten können [45, 46]. Der ICD-10-GM-Code für eine orthostatische Hypotonie lautet I95.1, während der Code für das POTS G90.80 ist.

Auch sollte ein Screening auf Hypermobilität erfolgen (Tab. 1), da diese bei einem relevanten Anteil der Betroffenen vorliegt [96] und auf ein hypermobiles Ehlers-Danlos-Syndrom (hEDS) hinweisen kann. Die Abklärung kann klinisch per Checkliste erfolgen (Tab. 1; [101]). hEDS wird auch in Verbindung mit einer kraniozervikalen Instabilität (CCI) gebracht, [138, 139] weist ähnliche Komorbiditäten wie ME/CFS auf und kann ein aggravierender Faktor bei Gelenk- und Muskelschmerzen sein. Zusätzlich sollte gerade bei Betroffenen mit Hypermobilität auch an das Vorliegen eines obstruktiven Schlafapnoe-Syndroms gedacht werden [140].

### Erfassung des Schweregrads von ME/CFS

ME/CFS kann zu schweren Funktionsbeeinträchtigungen führen, die das selbstständige Essen, Gehen oder selbstständige Körperhygiene für die Betroffenen unmöglich machen.

Es führt nicht selten zu erheblichen Beeinträchtigungen mit einem hohen Grad der Behinderung (GdB). Mit mehr als 60% der Betroffenen ist ein Großteil nicht mehr arbeitsfähig [141].

Der Schweregrad von ME/CFS ist in der Literatur gut dokumentiert. Die ME/CFS-Leitlinie der NICE [2], die Internationalen Konsensuskriterien (ICC) [73] und der Bericht des Instituts für Qualität und Wirtschaftlichkeit im Gesundheitswesen (IQWiG) aus Deutschland [15] unterscheiden vier Schweregrade: leichte, moderate, schwere und sehr schwere Ausprägung. Die Abgrenzungen zwischen den Gruppen sind nicht starr, sondern fließend und bieten einen Anhaltspunkt für den Einfluss des Schweregrads der Erkrankung auf die tägliche Funktionsfähigkeit. Schon eine „leichte“ Form der Erkrankung kann das mögliche Aktivitätslevel im Alltagsleben im Vergleich zu vor der Erkrankung um 50% verringern, was wiederum erhebliche Auswirkungen auf die soziale Integration und Teilhabe hat. Sehr schwer von ME/CFS Betroffene sind vollständig bettlägerig und pflegebedürftig, sehr stark reizempfindlich, können oft nicht mehr sprechen oder selbstständig Nahrung aufnehmen und keine Ärzt:innen mehr aufsuchen. (Eine Übersicht der vier Stufen unter: https://mecfs.at/ueber-me-cfs/#schweregrad).

Personen, die „leicht“ von der Erkrankung betroffen sind, sind oft nur in begrenztem Umfang arbeitsfähig, benötigen dabei erhebliche Anpassungen und sehen sich starken Einschränkungen gegenüber [141]. Etwa 25% der Betroffenen sind an Haus oder Bett gebunden [56]. Schwerstbetroffene müssen von allen Reizen abgeschirmt werden und sind vollständig pflegebedürftig – bis hin zur künstlichen Ernährung [142]. Die Belastung durch die Krankheit ist derart hoch, dass die durchschnittliche Lebensqualität der Erkrankten niedriger ausfällt als bei anderen gravierenden und stark limitierenden Krankheiten wie MS, Mukoviszidose, Diabetes mellitus, Epilepsie, AIDS oder Krebs [143, 144]. ME/CFS führt häufig zu Fehlzeiten in Schule und Ausbildung. Es wurde in anderen Ländern als häufigster Grund ungeklärter langer Schulfehlzeiten identifiziert. Nicht selten werden Familien deshalb mit Anfragen von Schul- oder Jugendämtern konfrontiert. Zudem beeinträchtigt ME/CFS nicht nur die Lebensqualität der Betroffenen erheblich, sondern wirkt sich auch stark auf die Lebensumstände der Angehörigen aus, die sich häufig ohne Unterstützung um die Betreuung und Pflege der Erkrankten kümmern müssen [1, 144–148].

In der Praxis wird zur Einstufung des Schweregrads von ME/CFS üblicherweise auf die Bell-Skala zurückgegriffen, die eine Einordnung des Gesundheitszustands in 10er-Schritten auf einer Skala von 0–100 ermöglicht. Die Bell-Skala wurde von David S. Bell speziell für ME/CFS entwickelt. 100 Punkte repräsentieren einen „gesunden Zustand“ und 0 Punkte eine schwerstbetroffene Person. Diese Skala hat sich sowohl in der Diagnosestellung, in der Verlaufsbeobachtung, als auch in der Forschung zu dieser Erkrankung bewährt: https://www.sgme.ch/bell-skala.

Bei der Beurteilung des Schweregrads ist es wichtig zu berücksichtigen, dass die gesundheitliche Verfassung bei ME/CFS häufig fluktuiert und eine einzige Untersuchung möglicherweise nicht das individuelle Spektrum der Betroffenen repräsentiert. Es empfiehlt sich daher, die Betroffenen nicht nur nach dem aktuellen Score auf der Bell-Skala, sondern nach dem besten und schlechtesten Wert im Krankheitsverlauf zu fragen. Zu beachten ist auch, dass die Anforderungen einer medizinischen Untersuchung zeitverzögert zu einer PEM führen können, so dass eine wiederholte Einschätzung auf der Bell-Skala sinnvoll sein kann.

Zukünftig könnte sich mit dem FUNCAP55 ein hilfreicher Fragebogen etablieren, der zu einer noch besseren Einschätzung der funktionellen Kapazität bei ME/CFS verhilft [149]. Der FUNCAP55 ist ein Instrument, das zur Beurteilung der funktionellen Leistungsfähigkeit bei ME/CFS entwickelt wurde. Damit kann der Schweregrad von ME/CFS berechnet und über den Krankheitsverlauf hinweg verfolgt werden. Die Schweizerische Gesellschaft für ME & CFS hat dazu einen praktikablen Schweregrad-Rechner konzipiert: https://sgme.ch/funcap55.

## Medizinische Versorgung/Therapie von ME/CFS

Auch nach Erhalt der Diagnose ME/CFS gibt es aktuell für Betroffene keine angemessene Versorgungsinfrastruktur im gesamten D‑A-CH-Raum und darüber hinaus [30, 33, 146, 150, 151]. Öffentlich finanzierte Betreuungsangebote, spezialisierte Disease-Management-Programme, spezialisierte Rehabilitationseinrichtungen oder Pflegeeinrichtungen für ME/CFS-Erkrankte fehlen vollständig. Zudem wird in den vorhandenen medizinischen Versorgungseinrichtungen die Erkrankung selbst und/oder die Schwere dieser Erkrankung häufig nicht oder nur unzureichend erkannt [30, 146].

Wird ME/CFS als psychische Erkrankung fehldiagnostiziert, folgen Therapieansätze wie Psycho- und aktivierende Bewegungstherapie, die für Betroffene schwerwiegende negative Konsequenzen haben können, [2, 25, 28, 35, 38, 39, 48, 71, 109] wenn sie in gewohnter Weise und ohne konsequente Anpassung an die Besonderheiten von ME/CFS sowie ohne die Berücksichtigung der PEM durchgeführt werden [33]. Dies gilt für alle etablierten psychotherapeutischen Verfahren gleichermaßen. Psychotherapie hat bei ME/CFS ausschließlich unterstützende Bedeutung und wird ohne kurative Zielsetzung durchgeführt. Schrittweise aktivierende Elemente sind schädlich und können den Zustand langfristig und irreversibel verschlechtern [2, 23, 26, 33, 36, 37, 101, 141]. Psychotherapeutische Interventionen, die den Respekt vor Belastungsgrenzen (im Rahmen von Pacing, s. unten) als Vermeidung fehldeuten und gegenüber den Patient:innen entsprechend problematisieren, sind im Falle von ME/CFS ebenfalls kontraproduktiv und unter Umständen schädlich [71].

Psychotherapeutische Unterstützung und Hilfestellungen bei der Krankheitsverarbeitung können andererseits wichtig sein, da die Betroffenen aufgrund ihrer massiven Einschränkungen und ihrer sozialen Folgen schwer belastet sind. Auch das nachfolgend beschriebene Pacing bedeutet eine enorme Herausforderung, die mit der Begleitung entsprechend geschulter klinischer Psycholog:innen, Psychotherapeut:innen, Ergotherapeut:innen [152] oder Physiotherapeut:innen oft besser bewältigt werden kann.

Wichtig sind auch eine entsprechende Behandlung von Komorbiditäten, die Unterstützung eines guten Schlafes (inkl. Schlafhygiene, Entspannungstrainings, Stresskontrolle) und die adäquate Behandlung von Schmerzen und Strategien im Umgang damit.

Physiotherapie zum Beispiel kann Schmerzen und Komorbiditäten bei Personen mit ME/CFS positiv beeinflussen. Außerdem können Patient:innen im Rahmen der Physiotherapie bei Pacingstrategien, der Regulation des autonomen Nervensystems und der Kreislaufaktivierung unterstützt werden [5, 33, 153–156]. Physiotherapeutische Maßnahmen müssen immer innerhalb der individuellen Energiegrenzen der Patient:innen gewählt und an das Pacing angepasst werden [5, 33, 153–156].

Auch speziell geschulte Diätolog:innen können ME/CFS-Patient:innen unterstützen, vor allem, wenn es zu diversen Nahrungsmittelunverträglichkeiten kommt und/oder bei schwerkranken Patient:innen zu Schluckproblemen. Die Exploration einer histaminarmen oder glutenfreien Diät kann hilfreich sein. Einem Gewichtsverlust muss gegebenenfalls gezielt durch ausreichende Kalorienzufuhr entgegengewirkt werden. In einzelnen Fällen ist eine Sondenernährung notwendig. Im Fall von schwer und schwerstbetroffenen ME/CFS-Patient:innen kommt auch der spezifisch geschulten Diplomkrankenpflege eine wichtige Rolle zu, da es in diesen Fällen oftmals eine „high-intensive home care“ braucht [56].

Bislang gibt es keine durch hochwertige wissenschaftliche Studien bestätigte Heilbehandlung für ME/CFS, was vermutlich auch auf die unzureichende finanzielle Unterstützung für klinische Forschung auf globaler Ebene zurückzuführen ist [6, 157]. Aus diesem Grund wäre auch die Prävention im Hinblick auf eine (Re‑)Infektion mit den im D‑A-CH-Raum am häufigsten vorkommenden Erregern wie SARS-CoV‑2, Influenza und Epstein-Barr-Virus (EBV) ein wichtiger Faktor.

Das Behandlungsprinzip für ME/CFS konzentriert sich auf umfassende Aufklärung über vorbeugendes Selbstmanagement, Therapieansätze, die auf die Linderung von Symptomen abzielen, und auf empathische psychosoziale Unterstützung als sekundäre Maßnahme [6]. Die Therapie beruht auf zwei Säulen:

### PACING: Individuelles Aktivitäts- und Energiemanagement

Pacing ist eine Form des Aktivitäts- und Energiemanagements, die ME/CFS-Erkrankten helfen soll, in den eigenen, teils sehr engen und von Tag zu Tag variierenden Leistungsgrenzen zu bleiben, um eine Zustandsverschlechterung durch PEM möglichst zu vermeiden [134, 158–160]. Der Ansatz wurde im Rahmen der ME/CFS-Forschung entwickelt und wird laut europaweitem Forschungsnetzwerk zu ME/CFS (EUROMENE) [1], CDC [25] und NICE [2]als wichtiger Teil des Krankheitsmanagements bei ME/CFS empfohlen. Eine auf PEM ausgerichtete Gesundheitsversorgung trägt signifikant dazu bei, Verschlechterungen bei den Betroffenen zu vermindern [161].

Ziel des Pacings ist es, den Gesamtzustand nach Möglichkeit zu stabilisieren und eine fortlaufende Verschlechterung durch wiederholte PEM zu verhindern. Mit Blick auf PEM gilt grundsätzlich: Keine Aktivierung über die individuell reduzierten Belastungsgrenzen hinaus [161]. ME/CFS-Betroffene müssen dazu lernen, ihren Tagesablauf mit allen Aktivitäten möglichst gut an ihre Leistungsgrenzen anzupassen. Routinen oder Hilfsmittel wie Pulsuhren können dabei hilfreich sein, ersetzen aber nicht die Einübung von Selbstbeobachtung und einer verlässlichen Körperwahrnehmung [71]. Auch der ME/CFS-Patient:innen-Leitfaden zur Vermeidung von PEM kann unterstützend und aufklärend sein: https://www.omf.ngo/wp-content/uploads/2019/09/PEM-Avoidance-Toolkit-Deutsch.pdf. Zusätzlich hat die Schweizerische Gesellschaft für ME & CFS eine hilfreiche Patient:innen-Broschüre zu Pacing erstellt: https://cdn.sgme.ch/pdf/Pacing-Broschuere.pdf.

Allerdings kann Pacing bei ME/CFS-Patient:innen mit einem sehr hohen Schweregrad kaum oder gar nicht umgesetzt werden, da bereits grundlegende und lebensnotwendige Tätigkeiten wie Essen oder minimale Bewegungen zu einer Zustandsverschlechterung führen können.

Es ist ausdrücklich kein Ziel von Pacing, die Leistungsgrenzen schrittweise zu steigern oder zu erweitern, wie es das Ziel von aktivierenden Ansätzen ist. Demnach sind z. B. Behandlungen in Rehabilitationskliniken mit Aktivierungstherapien, die sich auf schrittweise gesteigerte Übungsanforderungen als therapeutisches Mittel stützen und bei anderen chronischen Erkrankungen bewährt sind, nicht mit ME/CFS vereinbar. Eine Alternative können – je nach Schweregrad der individuellen Erkrankung – Maßnahmen wie ergo- und physiotherapeutische Hausbesuche sowie Teletherapie unter der Berücksichtigung von PEM sein.

### Symptomatische Linderung der begleitenden klinischen Problematik

Tab. 2 listet Publikationen auf, die orientierende Behandlungsvorschläge liefern, da es hierfür bereits erste schwache Evidenzen gibt.

**Tab. 2:** Publikationen, die einen orientierenden Überblick über Behandlungsoptionen liefern, da es für diese bereits erste, schwache Evidenz gibt

Übersichtsartikel	Zusammengefasster Überblick über Therapieoptionen (alle Angaben gelten nur, wenn keine Kontraindikationen vorliegen)
Renz-Polster und Scheibenbogen, 2022 [6]	*PEM:*Pacing
	*Orthostatische Dysfunktion*: Vermehrtes Trinken, v. a. vor dem ersten morgendlichen Aufrichten, erhöhte orale Salzzufuhr/Elektrolytlösungen, Stützstrümpfe hoch/Leibbinden. Bei ungenügendem Ansprechen „off-label“-Medikamente: Kardioselektive β‑Blocker/Ivabradin (cave: Blutdrucksituation), Pyridostigmin, Fludrokortison, Midodrin. Immer eine Gratwanderung zwischen Wirkung und Verträglichkeit
	*Schlafstörungen*: Melatonin 2–5 mg z. B. in retardierter Form, Antihistaminika der ersten Generation, Tryptophan, auch niedrig dosierte Antidepressiva (z. B. Trazodon) können helfen
	*Schmerzen*: Behandlung nach dem Prinzip der multimodalen Schmerztherapie (aber Beachtung von PEM), evtl. Pregabalin oder Low-Dose-Naltrexon (LDN). Kein Amitriptylin bei Tachykardie und POTS
	*Infektionskontrolle*: Behandlung einer möglichen Aktivierung von Viren, Immunglobuline bei IgG-Mangel
	*Sekundäre psychische Probleme (z.* *B. Depression, Ängste)*: Psychotherapeutische Unterstützung lt. Grande et al. 2023 [71]
	*Ernährung und Nahrungsergänzungsmittel*: Proteinreiche Ernährung mit ausreichend ungesättigten Fettsäuren z. B. Omega 3. Ausgleich eines Eisen‑, Folsäure‑, Vitamin D- und/oder Vitamin B12-Mangels, evtl. Therapieversuch zur Unterstützung der Mitochondrienfunktion mit D‑Ribose, Carnitin, Coenzym Q10, N‑Acetyl-Cystein (NAC), Nikotinamidadenindinukleotid (NADH), Magnesium, Selen und/oder Zink zur Unterstützung des Energiestoffwechsels oder der Redox-Balance. Schwerkranke Patient:innen sind teilweise auf pürierte Kost oder hochkalorische Trinknahrung angewiesen, sehr schwer Erkrankte müssen per Jejunalsonde ernährt werden
	*Aktive Allergie, Mastzellüberaktivität oder MCAS*: H1- und H2-Antihistaminika, eventuell ergänzt um einen Mastzellstabilisator wie Cromoglicinsäure oder Ketotifen
Charité Berlin: Empfehlungen für die Behandlung von Patient:innen mit postinfektiösem Syndrom/ME/CFS (G93.3G)	https://cfc.charite.de/weiterbildung/, https://mecfs.at/aerztinnen/#fortbildungen, einloggen bei DocCheck notwendig
	Informationen zu Schlafstörungen, neuropathischen Schmerzen, „off-label“-Therapien mit Mestinon (Pyridostigmin), Low-Dose Aripiprazol (LDA), Low-Dose Naltrexon (LDN), Ernährung und Nahrungsergänzungsmittel
Empfehlungen für die Behandlung von Patient:innen mit schwerem postinfektiösen Syndrom/ME/CFS (G93.3G)	https://cfc.charite.de/weiterbildung/, https://mecfs.at/aerztinnen/#fortbildungen, einloggen bei DocCheck notwendig
	Informationen zum Therapieversuch bei akutem Krankheitsschub (PEM), welcher oft nach Überanstrengung oder Infekt ausgelöst wird
	Informationen zur Therapie Schwerstbetroffener [56]
Das EUROMENE Consensus Protokoll [1]	Empfehlungen für eine nicht-medikamentöse Behandlung zur Linderung von ME/CFS-Symptomen in Bezug auf: PEM, Schmerzen, Schlaf, Ernährung und weitere unterstützende Maßnahmen
	Empfehlungen für eine medikamentöse Behandlung zur Linderung von ME/CFS-Symptomen in Bezug auf: Schmerzen, Schlaf, Dysautonomie (v. a. POTS), Allergien/Inflammationen, Nahrungsergänzungsmittel
Joseph et al., 2022 [162]	Mestinon (Pyridostigmin)
Seton et al., 2024 [117]	*PEM*: Pacing
	*Antivirale Therapie und Immunmodulatoren*: Nukleosid-Analoga, Immunmodulatoren
	*Inflammation*: Low-Dose Naltrexon (LDN), Mastzellstabilisatoren
	*Autoimmunität*: Immunsuppression, Immunadsorption
	*Immundefizienz*: Intravenöse Immunglobuline (IVIG)
	*Zelluläre, metabolische Abweichungen*: Antioxidantien, hochenergetische Verbindungen und Substrate des TCA-Zyklus, Mitochondrien-modulierende Nährstoffe
	*Gastrointestinale Dysfunktionen*: Probiotika
	*Neurologische Dysfunktionen*: SSRIs, SNRIs, Lisdexamfetamine, Nahrungsergänzungsmittel, Fludrocortison, siehe auch Inflammation
	*Neuroendokrine Dysfunktionen*: Hormone je nach Mangel (Low-Dose Hydrocortison, DHEA, Triiodothyronine)
Bested und Marshall, 2015 [28]	*PEM:* Pacing
	*Fatigue:* Stimulierende Medikamente nur, um Patient:innen bei außergewöhnlichen und potenziell anstrengenden Ereignissen zu helfen (z. B. Hochzeit)
	*Schlafstörungen:* Schlafhygiene, diverse Medikamente (Tab. 4)
	*Schmerzen:* Nicht-medikamentöse und medikamentöse (Tab. 5) Optionen werden aufgezählt (wichtig: mit nur einem Zehntel bis einem Viertel der üblichen Dosis starten bei Patient:innen, die empfindlich auf Medikamente reagieren)
	*Kognitive Dysfunktionen*: Pacing, Erinnerungsbuch
	*Management von sekundärer Depression, Angst und Stress*: Psychotherapie, falls Medikation notwendig, dann mit der niedrigmöglichsten Dosierung starten und langsam hochdosieren
	*Orthostatische Intoleranz*: Nicht-medikamentös: gesteigerter Salzkonsum, Elektrolytsäfte, viel Trinken, hohe Kompressionsstrümpfe, langes Stehen vermeiden; falls kein ausreichendes Ansprechen medikamentös: Fludrocortison, Midodrin und niedrigdosierte β‑Blocker
	*Gastrointestinale und urogenitale Probleme*: Abklärung (Nahrungsmittelunverträglichkeiten Dysbiosis, Leaky Gut), ausgewogene Ernährung mit wenigen Fermentable Oligo-Di-Monosaccharides and Polyols (FODMAPs), evtl. Nahrungsergänzungsmittel
	*Erreger-Reaktivierung*: Antibiotika, antiparasitäre oder antivirale Therapien können hilfreich sein
	*Allergien*: H1-Blocker
Rivera et al., 2019 [163]	Allgemeine Maßnahmenübersicht: Pacing, Mito-protektive Diäten (z. B. Intervallfasten oder ketogene Diät), Nahrungsergänzungsmittel, Probiotika
	*Schmerzen*: NSARs
	*Schlaf*: Trizyklische Antidepressiva, SSRIs (auch bei neuropathischen Schmerzen)
	*Virusreaktivierung und PEM*: Antivirale Medikamente
	*Inflammation:* Monoklonale Antikörper
	*Inflammation und hauptsächlich körperliche Fatigue:* Hydrocortison, Fludrocortison
ME/CFS Mayo Clinic Proceedings, 2023 [95]	*PEM:* Pacing, Health Tracker/Wearables oder Symptom-Tagebücher
	*Pathologische Fatigue*: Pacing, LDN, LDA, entzündungshemmende Diäten, Nahrungsergänzungsmittel bei Defiziten
	*Schlafstörungen*: Melatonin, Trazodon, Suvorexant, Doxepin/trizyklische Antidepressiva, Gabapentin/Pregabalin
	*Kognitive Dysfunktionen*: Tagebuchführung, Gedächtnisstützen, Ergotherapie, LDN, LDA, vorsichtiger Einsatz von Stimulanzien
	*Orthostatische Intoleranz*: Flüssigkeit/Elektrolyte/Kompression, Fludrocortison, Midodrin, Propranolol, Pyridostigmin, Guanfacin (Auswahl nach Subtyp des POTS oder Kippvitalzeichen richten)
	*Schwindel/Benommenheit:* POTS-Abklärung und entsprechende Therapie, vestibuläre Therapie
	*Muskel- oder Gelenksschmerzen*: Schmerzmittel, Duloxetin, Milnacipran, Pregabalin, Gabapentin, trizyklische Antidepressiva, LDN
	*Neuropathie*: Pregabalin, Gabapentin, trizyklische Antidepressiva, Kompressions- oder Korsetttherapie
	*Licht‑/Reizempfindlichkeit*: Kopfhörer mit Geräuschunterdrückung, getönte Brillen, Verringerung der Exposition, LDA
	*Gastrointestinale Symptome*: Entzündungshemmende Diäten, kleine Mahlzeiten, Pro‑/Synbiotika, Antihistaminika, Ballaststoffe
Bateman Horner Center: ME/CFS Guidebook	https://batemanhornecenter.org/education/mecfs-guidebook/
US ME/CFS Clinician Coalition, ME/CFS TREATMENT RECOMMENDATIONS US ME/CFS Clinician Coalition, Version 1, February 20, 2021	https://mecfscliniciancoalition.org/clinical-management/
	Medikamentöse Therapie: orthostatischen Dysfunktionen, Schlafstörung, kognitiver Dysfunktion, Fatigue, Schmerz, Immundysfunktionen, Small Intestinal Bacterial Overgrowth (SIBO)
	Nicht-medikamentösen Therapie: PEM, orthostatische Dysfunktionen, Schlafstörung, gastrointestinale Probleme, kognitive Beeinträchtigungen, Schmerzen

Dennoch handelt es sich weiterhin oft um „off-label“-Therapien, weshalb eine gute Aufklärung und Verschreibung durch einen Arzt oder eine Ärztin wichtig ist. Zudem müssen Nebenwirkungen, Wechselwirkungen und Kontraindikationen streng beachtet werden.

Wichtig ist es auch, das Vorliegen von Nahrungsmittelunverträglichkeiten genau zu untersuchen und z. B. im Falle von einer Laktoseunverträglichkeit laktosefreie Medikamente zu verschreiben. Auch können sich Nahrungsmittelunverträglichkeiten im Laufe der Erkrankung entwickeln.

Tab. 2 ist nicht als abschließend zu betrachten und nennt eine Auswahl an Publikationen, die sich mit diesem Thema beschäftigen, um einen Überblick zu geben. Wenn es mehrere Publikationen zu ME/CFS von der gleichen Organisation gab wie z. B. die ME/CFS Mayo Clinic Proceedings, wurde nur die mit dem aktuellsten Datum verwendet.

Die Therapie der symptomatischen Linderung der begleitenden klinischen Problematik stellt die Behandelnden auf dem Gebiet ME/CFS vor die große Herausforderung, die richtige Wahl aus der Übersicht der Möglichkeiten zu treffen. Eine zusätzliche Herausforderung ist, dass die meisten der Medikamente „off-label“ sind und dass einige der Medikamente wiederum vorausgehende Tests erfordern. Immer sind Kontraindikationen, Wechsel- und Nebenwirkungen zu beachten sowie die spezifische Dosierung bei den ME/CFS-Patient:innen.

Aus diesem Grund empfehlen wir in diesem D‑A-CH-Konsensus in einem ersten Schritt den Fokus auf folgende Symptomkomplexe und Medikamente/Therapien zu legen, die auch in Tab. 2 repetitiv vorkommen und damit mehrfach in den Übersichtsarbeiten erwähnt werden (siehe auch aktuelle Fortbildungen der Charité – Universitätsmedizin Berlin https://pcn.charite.de/fortbildung/).

Alle Empfehlungen gelten nur, wenn keine Kontraindikationen bestehen (Infobox 1).

#### Infobox 1 Empfehlungen von aktuellen Therapieoptionen, wenn keine Kontraindikationen bestehen

- *Adäquate Behandlung aller Symptome und der Komorbiditäten unter Beachtung von PEM*: Pacing first
- *PEM*: Pacing, Wearables, Aktivitäts‑/Symptom-Tagebücher.
- *Schlaf/Entspannung*: Entspannungstechniken, retardiertes Melatonin, H1-Antihistaminika der ersten Generation, z. B. Diphenhydramin, niedrig dosierte Antidepressiva (ein Zehntel bis ein Viertel der normalen Dosis: z. B. Mirtazapin, Trimipramin, Doxepin; kein Amitriptylin bei Tachykardie/POTS).
- *Orthostatische Intoleranz (OI) (POTS und OH)*: Mind. 3l Flüssigkeit/Tag trinken, v. a. auch Elektrolytsäfte, erhöhte Salzzufuhr, Stützstrumpfhose/Bauchbinde, Medikation: z. B. Ivabradin/Nebivolol [164], Mestinon, Fludrocortison, Midodrin je nach Symptomkomplex. Eventuell Volumensubstitution durch physiologische Kochsalzlösung.
- *Schmerzen:* Vorsichtige manuelle Therapie oder Physiotherapie, transkutane Nerven‑/Vagus-Stimulation, Medikation: z. B. Paracetamol/Ibuprofen/Metamizol, Gabapentin/Pregabalin, LDN je nach Schmerzsymptomatik, Involvierung der Schmerzmedizin.
- *Restless-legs-Symptomatik*: Versuch mit Dopaminagonist Rotigotion als Pflaster (1–3 mg/24 h)
- *Mastzellüberaktivität/MCAS:* H1 und H2-Blocker, eventuell unterstützt durch Mastzellstabilisatoren wie Ketotifen und Cromoglicinsäure.
- *Kognitive Dysfunktionen:* Je nach Befunden indirekte Therapie der kognitiven Dysfunktion über Symptomlinderung der Mastzellüberaktivität/MCAS und/oder OI sowie Pacing. LDN, LDA.
- *Nahrungsergänzungsmittel: *Zur Unterstützung des Mitochondrienstoffwechsels und/oder zum Ausgleich von Defiziten.
- *Neuroendokriner Hormonausgleich* bei Defiziten.
- *„Off-label“-Medikamente für Symptomenkomplexe*: Mestinon (POTS, Fatigue, PEM), [162] LDN (Fatigue, kognitive Dysfunktion, PEM, Schmerzen), [165, 166] LDA (Fatigue, kognitive Dysfunktion, PEM, Reizüberempfindlichkeit), [167] N‑Acetylcystein (NAC) und evtl. Guanfacin (Fatigue, kognitive Dysfunktion, PEM), [168] neuromodulatorische Medikamente, Medikamente bei Durchblutungsstörungen.
- *Bei Bedarf Unterstützung durch spezifisch geschulte* Diätolog:innen, Ergotherapeut:innen, klinische Psycholog:innen, Psychotherapeut:innen, Physiotherapeut:innen und diplomierte Krankenpflege.
- *Im Falle von Schwerstbetroffenen* braucht es eine „high-intensive home care“, in diesem Fall ist es wichtig, dass den Patient:innen und pflegenden Angehörigen entsprechend geschultes Gesundheitspersonal (z. B. für die Jejunalsondenpflege und Ernährung) und Hilfsmittel (von Antidekubitus-Matratzen bis zu elektrischen Rollstühlen mit Liegefunktion sowie zukünftige Möglichkeiten des Telemonitorings) zur Verfügung gestellt werden.

In einem weiteren Schritt kann diese Liste durch Medikamente und therapeutische Maßnahmen erweitert werden, die sich nach und nach im Rahmen von Studien als vorteilhaft erweisen, siehe auch „Fighting Post-Covid and ME/CFS – development of curative therapies“ von Scheibenbogen et al. 2023 [169]. Hier ist z. B. die Immunadsorption [170] zu nennen oder der Wirkstoffkandidat BC007, welcher sich gerade in einer klinischen Phase II Studie befindet. Wie weiter oben beschrieben, haben Patient:innen öfters funktionelle Antikörper gegen G proteingekoppelte Rezeptoren mit noch nicht gänzlich geklärter Relevanz für das Therapiemanagement. Diese könnten von diesen Therapien eventuell profitieren. Auch beschrieben wird dort hyperbare O2-Therapie (HBOT) [171]. Ein weiterer wichtiger Aspekt, welcher im Rahmen von Studien gesehen wurde, besteht in der Störung der Mikrozirkulation durch Mikrothromben [79–82]. Hier gibt es erste Hinweise aus Einzelstudien zu Therapieversuchen mit Antikoagulantien und Rheologika wie z. B. Sulodexide [172], und Vericiguat (Phase II Studie: https://clinicaltrials.gov/study/NCT05697640).

Es ist jedoch bei allen invasiven Maßnahmen neben den generellen Risiken zu bedenken, dass sich der Gesundheitszustand der Patient:innen durch eine hierdurch ausgelöste PEM auch verschlechtern kann. Dies gilt auch dann, wenn die Maßnahme erfolgreich angewandt wurde. Studien müssen auch im Hinblick darauf betrachtet werden, ob bei der Durchführung Rücksicht auf die besonderen Bedürfnisse dieser Patient:innengruppe (z. B. hinsichtlich Belastung, Einzelzimmer, Geräusch- und Lichtempfindlichkeit) genommen wurde.

Die ME/CFS-Forschung ist angesichts der hohen Zahl an Betroffenen und der erheblichen Krankheitslast international dramatisch unterfinanziert [173, 174]. Bisherige Forschungsinitiativen wurden vor allem durch private Gelder und Stiftungen ermöglicht. Dringend notwendig ist weitere Forschung, die sich z. B. damit beschäftigt, einfach messbare Indikatoren und Therapieoptionen zu identifizieren. Sinnvoll wären hierbei grenzüberschreitende Kooperationen der Forschungsinstitutionen, bei denen alle Forschenden einheitliche Diagnosestandards und eine einheitliche Unterscheidung der ME/CFS-Schweregrade zugrunde legen und anwenden.

Derzeit ist die Prognose der Erkrankung schlecht, nicht nur weil eine kurative Therapie fehlt, sondern auch wegen des oft großen zeitlichen Abstands zwischen dem Einsetzen der Symptome und der Diagnosestellung bzw. medizinischen Versorgung („diagnostic delay“) [175].

## Soziale Absicherung von ME/CFS-Erkrankten

Neben den fehlenden medizinischen Strukturen sind auch die Strukturen im Sozialsystem nicht auf ME/CFS-Betroffene ausgerichtet. Obwohl ein Großteil der Betroffenen nicht arbeitsfähig und ein Viertel sogar bettlägerig und pflegebedürftig ist [87–89], gibt es gravierende Probleme bei der behördlichen Anerkennung von Berufsunfähigkeitspension, Pflegestufe oder Behindertengrad und den damit verbundenen Unterstützungsleistungen. Trotz verbindlicher ICD-Kodierung von ME/CFS (ICD-10 G93.3) ist die Erkrankung meist nicht ausreichend be- und anerkannt [31]. Damit wird auch der Schweregrad der Erkrankung oft falsch eingeschätzt.

Aus Erfahrungen und Umfragen der ÖG ME/CFS unter Betroffenen sind für die Anerkennung der Schwere und Komplexität von ME/CFS und der damit verbundenen Einschränkungen in den meisten Fällen langjährige Gerichtsverfahren notwendig – oft auch ohne Erfolg. In vielen Fällen wird die Diagnose ME/CFS nicht in sozialrechtliche Gutachten übernommen oder ME/CFS wird in Diagnosen umgewandelt, die im Bereich der psychischen Erkrankungen liegen. Betroffene erhalten in diesen Fällen eventuell Anspruch auf Unterstützung, dieser ist aber bei einer Einstufung mittels psychischer Diagnose mit Auflagen verbunden, die dem Zustand der Betroffenen schaden. Die Situation der Betroffenen ist daher zusätzlich zur mangelnden medizinischen Versorgung auch in Bezug auf Existenzsicherung und soziale Absicherung erschreckend schlecht.

Viele Kinder und Jugendliche mit ME/CFS fehlen krankheitsbedingt regelmäßig in Schule, Ausbildung oder Studium, oft können sie den Regelunterricht gar nicht mehr wahrnehmen. In Studien aus unterschiedlichen Ländern wurde ME/CFS als häufiger Grund für lange Schulfehlzeiten gut belegt, [176–181] darunter auch die MUC-CFS-Studie aus Deutschland [182]. Dringend notwendig ist ein angemessener Nachteilsausgleich, der auf Basis eines ärztlichen Attests beantragt werden sollte und bestmöglich an die räumlichen und personellen Ressourcen der Heimatschule, des Ausbildungsplatzes und/oder der Universität angepasst sein sollte. Eine persönliche Abstimmung zwischen medizinischem Personal und Lehrkräften kann außerordentlich hilfreich sein. Informationen für Lehrkräfte finden sich englischsprachig auf der Webseite der CDC zu ME/CFS (https://www.cdc.gov/me-cfs/me-cfs-children/factsheet-educational-professional.html) oder deutschsprachig z. B. auf der Webseite der Elterninitiative für ME/CFS-kranke Kinder und Jugendliche München e. V. (https://www.mecfs-kinder-muc.de/me-cfs-und-jetzt/schule-lehrkraefte/). Viele betroffene Familien werden wegen der Schulfehlzeiten mit Schul- und/oder Jugendämtern konfrontiert und benötigen dort ME/CFS-Expertise, die leider noch häufig fehlt. Gerade für chronisch kranke Kinder und Jugendliche ist eine bestmögliche akademische Ausbildung von großem Wert, da körperlich anstrengende Berufe evtl. krankheitsbedingt nicht angestrebt werden können.

Eine Patient:innen-Umfrage der Schweizerischen Gesellschaft für ME & CFS zeigt außerdem, dass die mangelnde Sensibilisierung der Rentenversicherungen auf PEM ein großes Problem darstellt. Die meisten Rentenantragsteller:innen mit ME/CFS geben an, durch Überanstrengung während des Rentenverfahrens eine Zustandsverschlechterung erlitten zu haben. Bei mehr als einem Fünftel war diese schwer und irreversibel. Rentenverfahren müssen deshalb häufig vorzeitig abgebrochen werden. Kommt das Verfahren zum Abschluss, werden über drei Viertel der Anträge abgelehnt [183].

In der deutschen APAV-ME/CFS-Studie betonten die Betroffenen und ihre Angehörigen, dass nur durch die Anerkennung von ME/CFS als schwere Multisystemerkrankung durch die Ärzteschaft und die für sie zuständigen Personen innerhalb des Gesundheits- und Sozialsystems ihre Versorgung und soziale Absicherung gewährleistet werden könne. Sie forderten daher neben speziellen Fortbildungen und Schulungen für diese Personenkreise auch einen interdisziplinären Austausch und eine interdisziplinäre Zusammenarbeit aller beteiligten Professionen. Zudem betonten sie, dass insbesondere bei ME/CFS-Erkrankten mit schweren bzw. schwersten Einschränkungen nicht der Empowerment-Ansatz im Vordergrund stehen sollte, sondern ein auf ME/CFS zugeschnittenes Case-Management, dessen Ziel es ist, die Patient:innen in allen Bereichen des täglichen Lebens zu unterstützen, gerade weil sie eben nicht mehr in der Lage sind, sich selbst zu helfen [184].

Gutachterliche Relevanz hat ME/CFS vor allem im Schwerbehindertenrecht sowie bei der sozialmedizinischen Leistungsbeurteilung der Erwerbsfähigkeit der gesetzlichen Rentenversicherung. Für die diesbezügliche ärztliche Begutachtung steht seit 2023 eine übersichtliche Anleitung von Scheibenbogen et al., 2023 zur Verfügung [185].
